# Adjusting the range of cell–cell communication enables fine-tuning of cell fate patterns from checkerboard to engulfing

**DOI:** 10.1007/s00285-023-01959-9

**Published:** 2023-09-07

**Authors:** Simon Schardt, Sabine C. Fischer

**Affiliations:** Center for Computational and Theoretical Biology, University of Würzburg, Würzburg, Germany

**Keywords:** Cell differentiation, Pattern formation, Mathematical modeling, Statistical mechanics, 34D20, 37N25, 92C15

## Abstract

During development, spatio-temporal patterns ranging from checkerboard to engulfing occur with precise proportions of the respective cell fates. Key developmental regulators are intracellular transcriptional interactions and intercellular signaling. We present an analytically tractable mathematical model based on signaling that reliably generates different cell type patterns with specified proportions. Employing statistical mechanics, We derived a cell fate decision model for two cell types. A detailed steady state analysis on the resulting dynamical system yielded necessary conditions to generate spatially heterogeneous patterns. This allows the cell type proportions to be controlled by a single model parameter. Cell–cell communication is realized by local and global signaling mechanisms. These result in different cell type patterns. A nearest neighbor signal yields checkerboard patterns. Increasing the signal dispersion, cell fate clusters and an engulfing pattern can be generated. Altogether, the presented model allows us to reliably generate heterogeneous cell type patterns of different kinds as well as desired proportions.

**Supplementary Information:**

The online version contains supplementary material available at 10.1007/s00285-023-01959-9.

## Introduction

Cell fate decisions play an essential role in establishing cellular function during development. In this process, previously indeterminate cells specify themselves into one of several different cell types. In many cases, there is a strong correlation between gene expression patterns and subsequent cell fate. Therefore, it is necessary to understand the dynamics of different genes to unravel the secrets of differentiation.

One prime example of this differentiation process is the differentiation towards neural and epidermal cells in *Drosophila*. Characteristically, epidermal cell progenitors express high levels of transmembrane protein Notch, whereas neural progenitors express low levels of the same (Heitzler and Simpson 1991; Sternberg 1993). A similar example is found in the inner cell mass (ICM) of the preimplantation mouse embryo. There, the transcription factors (TFs) NANOG and GATA6 have been identified as the earliest markers for the segregation of the ICM into epiblast and primitive endoderm cells, respectively (Mitsui et al. 2003; Schrode et al. 2014). Apart from the spatial cell fate distribution, the correct cell fate ratio is also of particular interest (Saiz et al. 2016, 2020; Schröter et al. 2015).

In mathematical models, cell fate decisions are often described by systems of ordinary differential equations (ODE) based on a gene regulatory network (GRN) (reviewed in Torii 2012). At the single cell level, toggle switches as models of interactions of two genes have been investigated in great detail. These represent mutual inhibition of two proteins (Cherry and Adler 2000), which in some cases are combined with auto-activation (Huang et al. 2007). As a result, three stable steady states arise with regard to gene expression that represent the different cell fates. It depends on the initial conditions which state a cell will be attracted to. At the tissue level, experimental studies hint towards the importance of paracrine signals with regards to differentiation (Nichols et al. 2009; Yamanaka et al. 2010).

Lateral interaction models have already found their way into the current research landscape. For the Delta-Notch signaling pathway, patterns of alternating cell types have been reconstructed (Collier et al. 1996). For the mouse embryo, models including cell–cell communication due to fibroblast growth factor signaling have been employed to create similar salt-and-pepper/checkerboard patterns (Bessonnard et al. 2014; Tosenberger et al. 2017). So far, these studies are concerned with an averaged nearest neighbor signal, i.e. cells do not communicate beyond their nearest neighbor. Further studies suggest that in fact cell fate patterning in the mouse embryo is the result of a complex interplay of cell signaling, cell division, cell sorting and apoptosis (Morris et al. 2010a, b; Nissen et al. 2017).

Mathematical modeling allows untangling the individual components and investigating their pattern formation potential. It was previously shown that cell division alone yields cell fate clusters (Liebisch et al. 2020). Simulations of cells sorting due to differential adhesion have been shown to generate engulfing patterns (Revell et al. 2019). This resembles the result of the minimization of the total contact energy (Emily and François 2007). Here, we focus on the potential of intercellular signaling. In addition to nearest neighbor signaling, we consider signaling that can reach further across a tissue. This builds upon previous ideas for *Drosophila* (Chen et al. 2014; Cohen et al. 2010; de Joussineau et al. 2003) as well as the mouse embryo (Raina et al. 2021; Stanoev et al. 2021).

Based on methods from statistical mechanics (Bintu et al. 2005a, b; Garcia et al. 2011), we derived a model describing the temporal development of the expressions of two genes. A generalized signal incorporates external influences on cell fate decisions. Performing a detailed stability analysis of the ODE system, we obtained necessary conditions in the form of a parameter interval to always generate a mixture of two different cell types in a tissue. Numerical simulations for an averaged nearest neighbor signal as well as a distance-based signal demonstrate the potential of our model to establish different spatial cell fate patterns ranging from checkerboard via clustering to engulfing patterns. To quantify the different resulting patterns, we employed individualized pair correlation functions (PCFs). A cell type proportion analysis revealed which proportions our model can create, but also which restrictions there are. Our work introduces an easy to control mathematical model for gene expression and our analysis results provide insight into signaling driven pattern formation and cell type proportioning.

## Protein interaction model

**Fig. 1:**
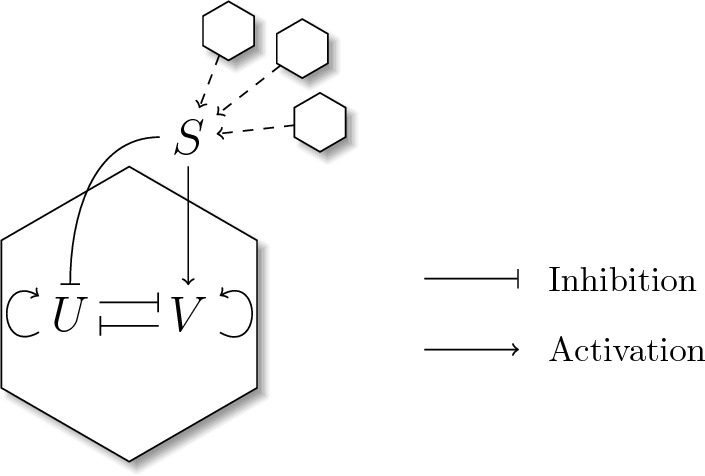
Illustration of the GRN considered in this study. Inside the cell, *U* and *V* inhibit each other. In addition to that, they activate themselves. The signal *S* is a factor that is influenced by the other cells in the tissue. It inhibits *U* and activates *V*

One of the aims of this study is to numerically simulate cell differentiation influenced by cell–cell communication. The cells will be fixed on an irregular grid regulating their concentration of key TFs internally, while also being influenced by surrounding cells. In this section, we will go through the details of transcriptional regulation based on a simple and generalized model. This will be followed by the derivation of TF binding probabilities based on the methods from Bintu et al. (2005a), Bintu et al. (2005b) and Garcia et al. (2011) which allow us to describe transcriptional regulation on the level of the DNA. Finally, this will be applied to a simple system of two different TFs *U* and *V* together with an external signal *S* describing the cell–cell communication. To this end, we consider a GRN characterized by the mutual inhibition of *U* and *V*, as well as their auto-activation and the signal *S* activating *V* and inhibiting *U* (Fig. 1). We consider this to be a general GRN for the cell fate decisions between two cell types, resembling similar GRNs from Huang et al. (2007) or Stanoev et al. (2021).

### Gene regulation

To describe the dynamical system underlying transcriptional regulation, we consider two basic assumptions:

1. Transcription determines the production of new protein.
2. Decay describes the lifetime of the protein.

These assumptions are translated into a generic ODE describing the nondimensionalized concentration $u=u(t)$ of the protein *U* over time:

1 $$\begin{aligned} \frac{du}{dt} = r_u p_u - \gamma _u u. \end{aligned}$$

The second term is the exponential decay with decay rate $\gamma _u$. The first term describes the rate of transcription of the corresponding gene. Here, $p_u$ denotes the probability that RNA polymerase (RNAP) is bound to the promoter of the gene that is associated with *U*. The production rate $r_u$ describes how much protein can be produced while RNAP is bound.

### Binding probability

Following (Bintu et al. 2005a, b; Garcia et al. 2011), we consider the different binding events of a GRN. However, we assume that the auto-activatory part of *U* is dominant, such that the base activity of the RNA polymerase will be neglected. This means that the production of *U* mainly depends on its binding close to its own promoter. Now the system can be in two different states. Either *U* is bound or it is not. First we count the number of possibilities how these states might arise. We divide our space into $\Omega$ different lattice sites, then *U* and *u* are related via $U = u \Omega$. The binomial coefficients yield the number of possible states

2 $$\begin{aligned}&\text {Number of unbound states:}{} & {} \frac{\Omega !}{U ! (\Omega - U)!} \end{aligned}$$

3 $$\begin{aligned}&\text {Number of bound states:}{} & {} \frac{\Omega !}{(U -1)! (\Omega - U + 1)!} \end{aligned}$$

Assuming different energies whether a protein is unbound $\varepsilon _u^{unbound}$ or bound $\varepsilon _u^{bound}$, the two states have total energies

4 $$\begin{aligned} \varepsilon ^{unbound}&= U \varepsilon _u^{unbound}, \end{aligned}$$

5 $$\begin{aligned} \varepsilon ^{bound}&= (U-1) \varepsilon _u^{unbound} + \varepsilon _u^{bound}. \end{aligned}$$

Using Boltzmann statistics, the energy of the two states enables us to describe the probability that the system is in either of these states via $e^{-\beta \varepsilon ^{unbound}}$ and $e^{-\beta \varepsilon ^{bound}}$. The partition function is given by the sum of all possible Boltzmann weights over every microstate, i.e.

6 $$\begin{aligned} Z_{total}&= \sum _{\text {microstates}} e^{-\beta \varepsilon _{\text {microstate}}} \end{aligned}$$

7 $$\begin{aligned}&= \frac{\Omega !}{U!(\Omega -U)!}e^{-\beta \varepsilon ^{unbound}} +\frac{\Omega !}{(U-1)!(\Omega -U+1)!}e^{-\beta \varepsilon ^{bound}} \end{aligned}$$

8 $$\begin{aligned}&= Z^{unbound} + Z^{bound}. \end{aligned}$$

Using the partition function, we are able to calculate the binding probability $p_u$ by the ratio of bound states $Z^{bound}$ and all states combined $Z^{unbound} + Z^{bound}$ as

9 $$\begin{aligned} p_u = \frac{Z^{bound}}{Z^{unbound}+Z^{bound}}. \end{aligned}$$

Assuming $\Omega \gg U$, we use the approximation $\frac{\Omega !}{(\Omega - U)!} \approx \Omega ^U$. We divide the numerator and denominator of (9) by $Z^{unbound}$ and define the energy difference $\Delta \varepsilon _u:= \beta (\varepsilon _u^{bound}-\varepsilon _u^{unbound})$ to obtain

10 $$\begin{aligned} p_u = \frac{Z^{bound}/Z^{unbound}}{1 + Z^{bound}/Z^{unbound}} = \frac{\frac{U}{\Omega }e^{-\Delta \varepsilon _u}}{1 + \frac{U}{\Omega }e^{-\Delta \varepsilon _u}}. \end{aligned}$$

For simplicity, we introduce the energy coefficient $\eta _u:= e^{-\Delta \varepsilon _u}$ and use $u=U/\Omega$ to get again the volume fractions. This leads to

11 $$\begin{aligned} p_u = \frac{\eta _u u}{1 + \eta _u u}. \end{aligned}$$

With Eq. (11) we have presented a binding probability solely based on the assumption that there is a difference between bound and unbound states. In general, TF-DNA interactions are much more complex and involve further effects like TF diffusion along the genome (Gerland et al. 2002). In our simplified model however, we only need statistical weights, e.g. $\eta _u$, which manipulate the likelihood of binding events. The terms “binding energy” and “energy difference” we use throughout this study should therefore be regarded with caution.

### Interactions

The crucial parts in transcriptional regulation are the interactions between constituents. In the following, we consider that an additional species *V* interacts with the promoter associated with *U*. This results in a system, with the following microstates:

**Table:** 

Binding event	Number of states
*U* unbound	$\frac{\Omega !}{U!V!(\Omega !-U-V)!}$
*V* unbound	
*U* bound	$\frac{\Omega !}{(U-1)!V!(\Omega !-U-V+1)!}$
*V* unbound	
*U* unbound	$\frac{\Omega !}{U!(V-1)!(\Omega !-U-V+1)!}$
*V* bound	
*U* bound	$\frac{\Omega !}{(U-1)!(V-1)!(\Omega !-U-V+2)!}$
*V* bound	

The binding energy differences remain as before with an additional factor for the interaction $\eta _{uv} = e^{-\Delta \varepsilon _{uv}}$. The binding probabilities for *U* and *V* are then given by

12 $$\begin{aligned} p_u = \frac{\eta _u u + \eta _u \eta _v \eta _{uv} uv}{1 +\eta _u u + \eta _v v + \eta _u \eta _v \eta _{uv} uv}. \end{aligned}$$

The advantage or disadvantage given by the interaction energy difference now determines the nature of the interaction. For $\eta _{uv}=1$, (12) can be simplified using factorization to obtain

13 $$\begin{aligned} p_u = \frac{\eta _u u + \eta _u \eta _v uv}{1 +\eta _u u + \eta _v v + \eta _u \eta _v uv} = \frac{\eta _u u (1 + \eta _v v)}{(1 + \eta _u u)(1 + \eta _v v)} = \frac{\eta _u u}{1 + \eta _u u}. \end{aligned}$$

The binding probability reduces to the case without interaction. Consequently, the cases where $\eta _{uv} \ne 1$ describe binding probabilities that are either lower or higher than the case with no interaction, hence

- $\eta _{uv} = 0 \quad \Leftrightarrow \quad \Delta \varepsilon _{uv} = \infty$: complete inhibition/blocking
- $$\eta _{uv} < 1 \quad \Leftrightarrow \quad \Delta \varepsilon _{uv} > 0$$: inhibition
- $$\eta _{uv} = 1 \quad \Leftrightarrow \quad \Delta \varepsilon _{uv} = 0$$: no interaction
- $$\eta _{uv} > 1 \quad \Leftrightarrow \quad \Delta \varepsilon _{uv} < 0$$: activation.

Case $\eta _{uv} = 0$ was listed separately, because it represents a special case of inhibition in which *U* and *V* cannot be bound at the same time. To improve readability, we use only lowercase letters in the following to denote both the concentrations in terms of ODEs and the actual proteins in the case of binding.

### Describing the cell fate decision between two fates

We imagine a system, where two antagonistic proteins *u* and *v* are the deciding factors for the decision of a cell’s fate. Both *u* and *v* are assumed to be dominantly auto-activating, such that the base activity of the RNAP can be neglected. Additionally, they will be influenced by an external signal *s*. Following the derivations from Sects. 2.2 to 2.3, this leads to a generalized binding probability for *u*

14 $$\begin{aligned} p_u = \frac{\eta _u u + \eta _u \eta _v \eta _{uv} u v + \eta _u \eta _s \eta _{us} u s + \eta _u \eta _v \eta _s \eta _{uvs} uvs}{1+\eta _u u + \eta _v v + \eta _u \eta _v \eta _{uv} u v + \eta _u \eta _s \eta _{us} u s + \eta _v \eta _s \eta _{vs} v s +\eta _u \eta _v \eta _s \eta _{uvs} uvs}\nonumber \\ \end{aligned}$$

Interchanging the letters *u* and *v* in (14) yields the binding probability $p_v$. As before, each summand describes a different binding event, using their individual coefficients $\eta _u$, $\eta _v$, and $\eta _s$ but also the interaction coefficients $\eta _{uv}$, $\eta _{us}$ and $\eta _{vs}$. Since we have three constituents *u*, *v* and *s*, we incorporate the possibility that all three of them are bound with interaction coefficient $\eta _{uvs}$.

We simplify Eq. (14) by putting it into biological context. We already mentioned the antagonistic nature of our proteins, i.e. *u* and *v* mutually inhibit each other. We use a blocking type of inhibition such that the promoter associated with *u* is not active as soon as *v* is bound in the vicinity of *u*’s promoter and vice-versa. We can also interpret this as *u* not being able to bind, if *v* is already bound, mathematically expressed via $\eta _{uv} = 0$. Finally, the external signal *s* influences *u* and *v* in different ways. It activates *v* by cooperatively binding with *v*, i.e. $\eta _{vs} \ge 1$. At the same time, *u* is inhibited by *s* and the cooperative binding of *u* and *s* leading to $\eta _{us}=0$ and $\eta _{uvs}=0$. We assume that for both promoters, the respective energy coefficients are equal. Summarizing the above, we obtain

15 $$\begin{aligned} \eta _{uv} = \eta _{us} = \eta _{uvs} = 0, \qquad \eta _{vs} \ge 1 \Longleftrightarrow -\Delta \varepsilon _{vs} > 0. \end{aligned}$$

Using (15) in (14), we obtain the binding probability for *u*

16 $$\begin{aligned} p_u = \frac{\eta _u u}{1 + \eta _v v(1+\eta _s \eta _{vs} s) + \eta _u u + \eta _s s}. \end{aligned}$$

Analogously, we can use the same approach for the binding probability of *v* to find

17 $$\begin{aligned} p_v = \frac{\eta _v v(1+\eta _s \eta _{vs} s)}{1 + \eta _v v(1+\eta _s \eta _{vs} s) + \eta _u u + \eta _s s}. \end{aligned}$$

With the expressions for (16) and (17), we can finally get back to the base model for transcriptional regulation (1). So far, we have described the transcriptional regulation of a single cell. However, by extending the system to *N* different cells, we can also describe a tissue of identically functioning cells. Combined, this leads to the following system of ODEs

18 $$\begin{aligned} \begin{aligned} \frac{du_i}{dt}&= r_u \frac{\eta _u u_i}{1 + \eta _v v_i(1+\eta _s \eta _{vs} s_i) + \eta _u u_i + \eta _s s_i} - \gamma _u u_i \\ \frac{dv_i}{dt}&= r_v \frac{\eta _v v_i(1+\eta _s \eta _{vs} s_i)}{1 + \eta _v v_i(1+\eta _s \eta _{vs} s_i) + \eta _u u_i + \eta _s s_i} - \gamma _v v_i, \qquad i = 1,...,N. \end{aligned} \end{aligned}$$

We note that so far, we have very little assumptions on the signal *s*. However, it should be noted that the signal is meant to be provided by the surrounding cells in the tissue. This means that the absorbed signals of each cell $s_i$ are provisionally considered as a generalized function of the expression values of all cells such that

19 $$\begin{aligned} {\varvec{s}}: {\mathbb {R}}^N \times {\mathbb {R}}^N \rightarrow {\mathbb {R}}^N: ({\varvec{u}},{\varvec{v}}) \mapsto {\varvec{s}}({\varvec{u}},{\varvec{v}}). \end{aligned}$$

Thus, Eq. (18) present a coupled system of ODEs.

## Steady state analysis

### Existence of steady states

In order to get a better understanding of our ODE system, we want to delve further into the resulting steady states of the system. This means, we consider

$$\begin{aligned} \frac{du_i}{dt} = 0 = \frac{dv_i}{dt}. \end{aligned}$$

Consequently, we get

20 $$\begin{aligned} \frac{\eta _u u_i}{1 + \eta _u v_i(1+\eta _s\eta _{vs}s_i) + \eta _u u_i + \eta _s s_i}&= \frac{\gamma _u}{r_u} u_i, \end{aligned}$$

21 $$\begin{aligned} \frac{\eta _v v_i(1+\eta _s\eta _{vs}s_i)}{1 + \eta _v v_i(1+\eta _s\eta _{vs}s_i) + \eta _u u_i + \eta _s s_i}&= \frac{\gamma _v}{r_v} v_i. \end{aligned}$$

When rearranging (20) and (21), we find two possible solutions for $u_i$ and $v_i$, respectively. These solutions are

22 $$\begin{aligned} u_i = {\left\{ \begin{array}{ll} 0 &{} \\ \frac{r_u}{\gamma _u} - \frac{1+\eta _v v_i(1 + \eta _s \eta _{vs} s_i) + \eta _s s_i}{\eta _u} \end{array}\right. } \text{ and }\quad v_i = {\left\{ \begin{array}{ll} 0 &{} \\ \frac{r_v}{\gamma _v} - \frac{1+\eta _u u_i + \eta _s s_i}{\eta _v(1 + \eta _s \eta _{vs} s_i)} \end{array}\right. }. \end{aligned}$$

The steady states of our ODE system have to fulfill (20) and (21) together. Thus, we look at every possibly combinations of pairs $u_i$ and $v_i$ from (22) to obtain four different steady states. For three of the steady states, we can get either no expression of *u* and *v* or high expression of one TF and none for the other:

23 $$\begin{aligned} u_i&= 0,{} & {} \quad v_i = 0 \end{aligned}$$

24 $$\begin{aligned} u_i&= \frac{r_u}{\gamma _u}-\frac{1+\eta _s s_i}{\eta _u},{} & {} \quad v_i = 0 \end{aligned}$$

25 $$\begin{aligned} u_i&= 0,{} & {} \quad v_i = \frac{r_v}{\gamma _v}-\frac{1+\eta _s s_i}{\eta _v(1+\eta _s \eta _{vs} s_i)}. \end{aligned}$$

These steady states share the lower bound 0. Additionally, a rough estimate for an upper bound is given by the ratios of reproduction and decay $r_u/\gamma _u$ and $r_v/\gamma _v$. For parameter combinations such that

26 $$\begin{aligned} \frac{r_u}{\gamma _u} \gg \frac{1}{\eta _u}, \quad \frac{r_v}{\gamma _v} \gg \frac{1}{\eta _v} + \frac{\eta _s}{\eta _v}s_i, \end{aligned}$$

the left hand sides of the inequalities provide a reliable estimate for the steady state values.

The fourth steady state is an oddity that arises by combining the non-zero solutions for $u_i$ and $v_i$ from (22). When combined, the corresponding variables $u_i$ and $v_i$ cancel out and we find the relation

27 $$\begin{aligned} \eta _v (1 + \eta _s \eta _{vs} s_i) = \eta _u \frac{r_u \gamma _v}{r_v \gamma _u}. \end{aligned}$$

This also leaves our system to be over-determined and the values of $u_i$ and $v_i$ cannot further be identified. However, by using (27) in the steady state solution $v_i \ne 0$ in (22), we obtain the following state:

28 $$\begin{aligned} u_i + \frac{r_u\gamma _v}{r_v\gamma _u} v_i = \frac{r_u}{\gamma _u} - \frac{1+\eta _s s_i}{\eta _u}. \end{aligned}$$

**Fig. 2:**
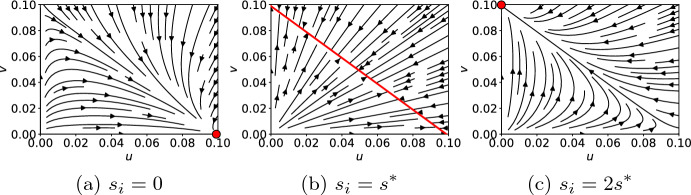
Streamline phase portraits of ODE system (18) for a single cell and three different values for $s_i$. Arrows show the path from the initial condition towards the respective steady states. States (24) and (25) are visualized as a single red dot, whereas the infinite steady states resulting from (28) are depicted as a red line

Isolating $s_i$ in Eq. (27) leads to a critical signal value

29 $$\begin{aligned} s^* = \frac{r_u \gamma _v \eta _u - r_v \gamma _u \eta _v}{r_v \gamma _u \eta _v \eta _s \eta _{vs}}, \end{aligned}$$

for which this steady state will always occur. This critical signal value $s^*$ contains all of the model parameters and resembles a difference of the statistical weights $\eta _u$ and $\eta _v$ normalized by the remaining model parameters. Furthermore, $s^*$ is responsible for a switching behavior in our system (Fig. 2). For values below or above $s^*$, a cell ends up in states (24) ($u^+v^-$) and (25) ($u^-v^+$), respectively. At exactly $s^*$, *u* and *v* move towards the straight line defined by (28) with no unique steady state. Altogether, we have successfully identified the relevant steady states (23)–(25) of our ODE system (18) as well as the condition to force a switch in the cell’s fate.

### Linear stability analysis

In the following sections, we investigate the steady states in further detail. We employ linear stability analysis to determine the parameter regime that allows us to find a desired steady state for the overall system. At the single cell level, we rule out (23), since it is not relevant to cell fate specification. At the tissue level, we distinguish between homogeneous and heterogeneous steady states. A homogeneous equilibrium state consists of cells of a single type only. This means that either all of the cells in the tissue are in state (24) ($u^+v^-$) or all of them are in state (25) ($u^-v^+$). Throughout this study, we aim for states of the tissue where two different cell types co-occur, i.e. heterogeneous equilibrium states. Therefore, we aim at excluding the homogeneous steady states. We follow the definition of linear stability for an ODE system

$$\begin{aligned} \frac{dx_i}{dt} = f(x), \qquad i = 1,...,N. \end{aligned}$$

We say, an ODE system is linearly stable in $x^*$, if its linearization matrix $L^{ODE} = f'(x^*)$ has only eigenvalues with negative real part. Using the *N*-dimensional identity matrix $I_N$, we can write the linearization matrix of (18) as

30 $$\begin{aligned} L^{ODE} = \begin{pmatrix} r_u A_{uu} - \gamma _u I_N &{}\quad r_u A_{uv} \\ r_v A_{uv} &{}\quad r_v A_{vv} - \gamma _u I_N \end{pmatrix}, \end{aligned}$$

Using the chain rule, the block matrices $A_{xy}$, $x,y \in \{u,v\}$ can be written in terms of the partial derivatives

31 $$\begin{aligned} A_{uu}&= \frac{\partial p_u}{\partial u} + \frac{\partial p_u}{\partial s}\frac{\partial s}{\partial u},&A_{uv}&= \frac{\partial p_u}{\partial v} + \frac{\partial p_u}{\partial s}\frac{\partial s}{\partial v}, \end{aligned}$$

32 $$\begin{aligned} A_{vu}&= \frac{\partial p_v}{\partial u} + \frac{\partial p_v}{\partial s}\frac{\partial s}{\partial u},&A_{vv}&= \frac{\partial p_v}{\partial v} + \frac{\partial p_v}{\partial s}\frac{\partial s}{\partial v}, \end{aligned}$$

where we define $\frac{\partial p_u}{\partial u}:= \left( \frac{\partial p_u}{\partial u_j,}(u_i,v_i,s_i)\right) _{i,j=1,...,N}$. The other block matrices are defined analogously. For our purposes, we only need to focus on the following derivatives

33 $$\begin{aligned} \frac{\partial }{\partial u_j}p_u(u_i,v_i,s_i)&= {\left\{ \begin{array}{ll} \frac{\eta _u(1+\eta _v v_i (1+\eta _s\eta _{vs}s_i) + \eta _s s)}{(1 + \eta _v v_i(1+\eta _s \eta _{vs} s_i) + \eta _u u_i + \eta _s s_i)^2}, &{} \qquad \text {if } i = j \\ 0, &{} \qquad \text {if } i \ne j \end{array}\right. } \end{aligned}$$

34 $$\begin{aligned} \frac{\partial }{\partial v_j}p_u(u_i,v_i,s_i)&= {\left\{ \begin{array}{ll} -\frac{\eta _v \eta _u u_i (1 + \eta _s \eta _{vs}s_i)}{(1 + \eta _v v_i(1+\eta _s \eta _{vs} s_i) + \eta _u u_i + \eta _s s_i)^2}, &{} \qquad \text {if } i = j \\ 0, &{} \qquad \text {if } i \ne j \end{array}\right. } \end{aligned}$$

35 $$\begin{aligned} \frac{\partial }{\partial u_j}p_v(u_i,v_i,s_i)&= {\left\{ \begin{array}{ll} -\frac{\eta _v \eta _u v_i (1 + \eta _s \eta _{vs}s_i)}{(1 + \eta _v v_i(1+\eta _s \eta _{vs} s_i) + \eta _u u_i + \eta _s s_i)^2}, &{} \qquad \text {if } i = j \\ 0, &{} \qquad \text {if } i \ne j \end{array}\right. } \end{aligned}$$

36 $$\begin{aligned} \frac{\partial }{\partial v_j}p_v(u_i,v_i,s_i)&= {\left\{ \begin{array}{ll} -\frac{\eta _v (1 + \eta _s \eta _{vs}s_i)(1 + \eta _u u_i + \eta _s s_i)}{(1 + \eta _v v_i(1+\eta _s \eta _{vs} s_i) + \eta _u u_i + \eta _s s_i)^2}, &{} \qquad \text {if } i = j \\ 0, &{} \qquad \text {if } i \ne j \end{array}\right. } \end{aligned}$$

37 $$\begin{aligned} \frac{\partial }{\partial s_i}p_u(u_i,v_i,s_i)&= -\frac{\eta _u \eta _s u_i (1 + \eta _v \eta _{vs}v_i)}{(1 + \eta _v v_i(1+\eta _s \eta _{vs} s_i) + \eta _u u_i + \eta _s s_i)^2} \end{aligned}$$

38 $$\begin{aligned} \frac{\partial }{\partial s_i}p_v(u_i,v_i,s_i)&= \frac{\eta _v \eta _s v_i (\eta _{vs} + \eta _u \eta _{vs}u_i - 1)}{(1 + \eta _v v_i(1+\eta _s \eta _{vs} s_i) + \eta _u u_i + \eta _s s_i)^2}. \end{aligned}$$

As usual, the eigenvalues of matrix $L^{ODE}$ are defined as the roots of the characteristic polynomial

39 $$\begin{aligned} \chi (\lambda ) = \det (L^{ODE}-\lambda I_{2N}), \end{aligned}$$

where $I_{2N}$ denotes the identity matrix in 2*N* dimensions. At first glance, this determinant seems impossible to calculate. However, when inserting the respective steady states, we are able to reduce the matrix tremendously.

### Excluding steady state (23)

In the following, we elaborate on how to exclude the first steady state (23) as stable solution for our ODE system (18). Without loss of generality, we assume $u_1 = 0 = v_1$.

This way, in row $N+1$ all entries but one of the matrix $L^{ODE}-\lambda I_{2N}$ become 0. The remaining entry with index $(N+1, N+1)$ is

40 $$\begin{aligned} r_v\frac{\partial }{\partial v_1}p_v(0,0,s_1) - \gamma _v - \lambda = r_v\eta _v \frac{1+\eta _s \eta _{vs} s_1}{1+\eta _s s_1} - \gamma _v - \lambda . \end{aligned}$$

Laplace expansion then enables us to write the determinant of the whole matrix as a product of (40) and the determinant of the remaining submatrix. Thus, it suffices to focus on the first eigenvalue given by

$$\begin{aligned} r_v\eta _v \frac{1+\eta _s \eta _{vs} s_1}{1+\eta _s s_1} - \gamma _v - \lambda {\mathop {=}\limits ^{!}} 0. \end{aligned}$$

This translates to the eigenvalue $\lambda$ being

$$\begin{aligned} \lambda = r_v\eta _v \frac{1+\eta _s \eta _{vs} s_1}{1+\eta _s s_1} - \gamma _v. \end{aligned}$$

Now, $$\lambda > 0$$ yields

$$\begin{aligned} \eta _v > \frac{\gamma _v}{r_v} \frac{1+\eta _s s_1}{1+\eta _s \eta _{vs} s_1}. \end{aligned}$$

Although the signal thus far has not been further specified, we propose a realistic physical representation by assuming $s_i \ge 0$. Furthermore, we consider an activation of *v* by the signal *s*, i.e. $\eta _{vs}>1$ and therefore, inequality

41 $$\begin{aligned} \eta _v > \frac{\gamma _v}{r_v} \end{aligned}$$

and consequently

42 $$\begin{aligned} -\Delta \varepsilon _v > \ln \left( \frac{\gamma _v}{r_v}\right) \end{aligned}$$

provide the necessary condition for instability. The exclusion of this steady state strengthens our focus on (24) and (25), which represent the two different cell types $u^+v^-$ and $u^-v^+$, respectively.

### Instability of tissue-wide homogeneous steady state (24)

With steady states (24) and (25), we aim to find a parameter region for which we achieve a heterogeneous steady state, i.e. we get a tissue with a mixture of cells in the two states. To this end, we derive conditions for instability of the homogeneous steady state. We start with state (24) and set $u_i = \frac{r_u}{\gamma _u}-\frac{1+\eta _s s_i}{\eta _u}$ and $v_i = 0$ for all *i*. Inserting these expressions into the derivatives (33)–(38) results in a simplification of $L^{ODE}$. Since (35) and (38) are zero for every *i*, *j*, the off-diagonal block matrix $A_{vu}={\varvec{0}}$. This means the determinant is given by the product of the determinants of the block matrices on the diagonal. Again, since (38) is zero, $A_{vv}$ becomes a diagonal matrix with diagonal entries

43 $$\begin{aligned} (A_{vv})_i = \frac{\eta _v(1+\eta _s\eta _{vs}s_i)}{1+\eta _u u_i + \eta _s s_i}, \qquad i = 1,...,N. \end{aligned}$$

Inserting $$u_i$$ yields

44 $$\begin{aligned} (A_{vv})_i = \frac{\gamma _u}{r_u} \frac{\eta _v}{\eta _u} (1+\eta _s\eta _{vs}s_i), \qquad i = 1,...,N. \end{aligned}$$

Using this, we determine *N* factors of the characteristic polynomial

45 $$\begin{aligned} \chi (\lambda )&= \det \left( r_u A_{uu}-(\gamma _u+\lambda )I_N\right) \det \left( r_v A_{vv}-(\gamma _v+\lambda )I_N\right) \end{aligned}$$

46 $$\begin{aligned}&= \det \left( r_u A_{uu}-(\gamma _u+\lambda )I_N\right) \left[ \prod _{i=1}^N \gamma _u\frac{r_v\eta _v}{r_u\eta _u}(1+\eta _s \eta _{vs} s_i) - \gamma _v - \lambda \right] \end{aligned}$$

and *N* eigenvalues are given by the second factor in (46). For instability, it is sufficient that only one of these is greater than zero. In other words, this results in the inequality

$$\begin{aligned} \gamma _u\frac{r_v\eta _v}{r_u\eta _u}(1+\eta _s \eta _{vs} s_i) > \gamma _v. \end{aligned}$$

After appropriate rearranging, we obtain

47 $$\begin{aligned} \eta _u < \eta _v \frac{r_v \gamma _u}{r_u \gamma _v}(1+ \eta _s\eta _{vs} \max _i s_i) \end{aligned}$$

as a sufficient condition for our parameters. At this point, the general case cannot be simplified further. Depending on the cell–cell interaction and therefore the incoming signal $s_i$, one can find an even more accurate description of this relation. Alternatively, we can formulate this condition in terms of energy differences as

48 $$\begin{aligned} -\Delta \varepsilon _u < -\Delta \varepsilon _v + \ln \left( 1+ e^{-\Delta \varepsilon _s-\Delta \varepsilon _{vs}} \max _i s_i\right) + \ln \left( \frac{r_v \gamma _v}{r_u \gamma _v}\right) , \end{aligned}$$

which allows us to see the maximum allowed deviation of the difference between $\Delta \varepsilon _u$ and $\Delta \varepsilon _v$. Keep in mind that for this condition, we only relied on the first *N* eigenvalues. In truth, this condition might be even more relaxed than what we derived.

### Instability of tissue-wide homogeneous steady state (25)

We set $u_i = 0$ and $v_i = \frac{r_v}{\gamma _v}-\frac{1+\eta _s s_i}{\eta _v(1+\eta _s \eta _{vs} s_i)}$. Using the same approach as before, we find that (34) and (37) are zero for all *i*, *j* and thus $A_{uv} = {\varvec{0}}$. In addition to that, we get a diagonal matrix for $A_{uu}$. For $u_i=0$, its diagonal entries are

49 $$\begin{aligned} (A_{uu})_i = \frac{\eta _u}{1+\eta _v v_i(1+\eta _s\eta _{vs}s_i)+\eta _s s_i}, \qquad i = 1,...,N. \end{aligned}$$

Inserting $$v_i$$ yields

50 $$\begin{aligned} (A_{uu})_i = \frac{\gamma _v}{r_v}\frac{\eta _u}{\eta _v}\frac{1}{1+\eta _s\eta _{vs}s_i}, \qquad i = 1,...,N. \end{aligned}$$

As before, this allows us to determine *N* factors of the characteristic polynomial

51 $$\begin{aligned} \chi (\lambda )&= \det \left( r_u A_{uu}-(\gamma _u+\lambda )I_N\right) \det \left( r_v A_{vv}-(\gamma _v+\lambda )I_N\right) \end{aligned}$$

52 $$\begin{aligned}&= \left[ \prod _{i=1}^N \gamma _v\frac{r_u}{r_v}\frac{\eta _u}{\eta _v}\frac{1}{1 + \eta _s \eta _{vs}s_i}-\gamma _u-\lambda \right] \det \left( r_v A_{vv}-(\gamma _v+\lambda )I_N\right) . \end{aligned}$$

We exploit again the instability condition that any eigenvalue must be positive and find the inequality

53 $$\begin{aligned} \eta _u > \frac{r_v \gamma _u}{r_u \gamma _v}\eta _v(1 + \eta _s \eta _{vs}s_i). \end{aligned}$$

This yields another condition for $\eta _u$. As before, it is necessary to fulfill this inequality for a single value $s_i$, i.e. the minimum of all possible signal values suffices in that regard. Hence, we get

54 $$\begin{aligned} \eta _u > \frac{r_v \gamma _u}{r_u \gamma _v}\eta _v(1 + \eta _s \eta _{vs}\min _i s_i). \end{aligned}$$

Again, we write this in terms of energy differences

55 $$\begin{aligned} -\Delta \varepsilon _u > -\Delta \varepsilon _v + \ln \left( 1+ e^{-\Delta \varepsilon _s-\Delta \varepsilon _{vs}} \min _i s_i\right) + \ln \left( \frac{r_v \gamma _u}{r_u \gamma _v}\right) . \end{aligned}$$

### Steady state summary

The stability conditions (48) and (55) define an interval

56 $$\begin{aligned} \Delta \varepsilon _{min}< -\Delta \varepsilon _u < \Delta \varepsilon _{max} \end{aligned}$$

with

57 $$\begin{aligned} \Delta \varepsilon _{min}&:= -\Delta \varepsilon _v + \ln \left( 1+ e^{-\Delta \varepsilon _s-\Delta \varepsilon _{vs}} \min _i s_i\right) + \ln \left( \frac{r_v \gamma _u}{r_u \gamma _v}\right) , \end{aligned}$$

58 $$\begin{aligned} \Delta \varepsilon _{max}&:= -\Delta \varepsilon _v + \ln \left( 1+ e^{-\Delta \varepsilon _s-\Delta \varepsilon _{vs}} \max _i s_i\right) + \ln \left( \frac{r_v \gamma _u}{r_u \gamma _v}\right) . \end{aligned}$$

**Fig. 3:**
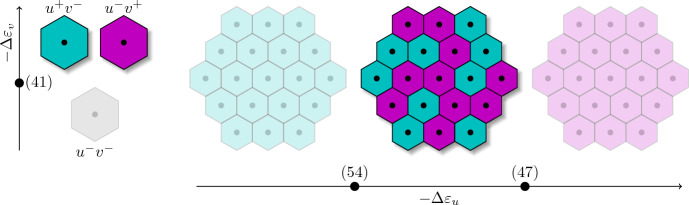
Illustration of the different steady states at the single cell level (left) and the tissue level (right). The states we are aiming for are highlighted with higher opacity. Nodes and their corresponding number on the axes reference the relevant equation for the transition from one state to another

The reproduction rates $r_u, r_v$ and decay rates $\gamma _u, \gamma _v$ shift this interval by $\ln \left( \frac{r_u \gamma _v}{r_v \gamma _u}\right)$. The length of the interval is determined by the minimum and maximum signal values combined with the associated energy differences $-\Delta \varepsilon _s$ and $-\Delta \varepsilon _{vs}$. The results of our stability analysis are summarized in Fig. 3. At the single cell level, we are able to exclude $u^-v^-$ cells using inequality (42). Therefore, at the tissue level, we can distinguish between three different states. The stability interval (56) yields the exact parameter regime for the transition of the homogeneous states to the heterogeneous ones. These elegant lower and upper bounds for $-\Delta \varepsilon _u$ incorporate every parameter in our ODE system (18). Finally, we know that the lower bound in (56) is associated with the homogeneous $u^-v^+$ state, whereas the upper bound is associated with the homogeneous $u^+v^-$ state. Therefore, we expect a monotonous increase in the number of $u^+v^-$ cells as the energy difference $-\Delta \varepsilon _u$ increases.

## Tissue organization

### Cell graph

**Fig. 4:**
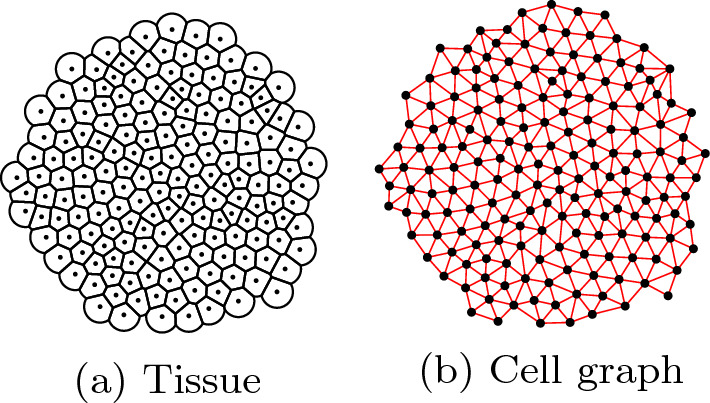
Visualization of a tissue with 177 cells (**a**) and its corresponding cell graph (**b**). Black lines represent the cell membranes. The cell centroids are shown as black dots in both pictures. Red lines represent the edges, which provide information about which cells are in contact with each other (color figure online)

Cells are represented by two-dimensional points in space with a fixed radius which is equal for all cells. The Delaunay cell graph provides a reliable indication of the neighborhood relationships of the cells (Schmitz et al. 2017). Therefore, we initialize our graph *G* using the Delaunay triangulation. If the Euclidean distance between two cells exceeds the sum of their two radii, then the edge is removed from *G*, i.e. only cells in direct contact with each other are connected via an edge in *G* (Fig. 4). Edge weights are collectively set to 1. We then define the cell distance $d_{ij}$ as the length of the shortest path between cells *i* and *j*. In this work, we chose to perform the simulations on a two-dimensional tissue for easier visualisation. However, the same approach has already been successfully applied in three spatial dimensions (Dirk et al. 2022).

### Pair correlation function

Cell differentiation patterns in our case are the result of two different cell types arising in a tissue. Patterns with the same condition have already been quantified using pair correlation functions (PCFs) (Binder and Simpson 2013). We use a similar approach to quantify our patterns with a PCF depending on the cell distances $d_{ij}$. The PCF relates the number of cell pairs of equal type to the random chance of picking two cells of equal type. Thus, it enables us to find accumulations of cell types at given distances towards each other. This requires counting different types of cell pairings for certain distances. In order to describe the PCF mathematically, we first introduce various sets. The set $S_k$ describes all the cell pairs (*i*, *j*) found at a distance $d_{ij}$. Similarly, $S^u_k$ denotes the set of all $u^+v^-$ cell pairs with distance *k*. Analogously, $S^v_k$ is defined for $u^-v^+$ cell pairs. Finally, the two sets $T^u$ and $T^v$ contain all $u^+v^-$ cells and $u^-v^+$ cells respectively. In mathematical notation, we write

59 $$\begin{aligned} S_k&= \left\{ (i,j)\in {\mathbb {N}}^2: d_{ij} = k,\, 1 \le i,j \le N\right\} , \end{aligned}$$

60 $$\begin{aligned} S^u_k&= \left\{ (i,j)\in S_k: i\text { and }j \text { are } u^+v^- \text { cells} \right\} , \end{aligned}$$

61 $$\begin{aligned} S^v_k&= \left\{ (i,j)\in S_k: i\text { and }j \text { are } u^-v^+ \text { cells} \right\} , \end{aligned}$$

62 $$\begin{aligned} T^u&= \left\{ i \in {\mathbb {N}}: i \text { is } u^+v^- \text { cell} \, 1 \le i \le N\right\} , \end{aligned}$$

63 $$\begin{aligned} T^v&= \left\{ i \in {\mathbb {N}}: i \text { is } u^-v^+ \text { cell}\, 1 \le i \le N\right\} . \end{aligned}$$

In the next step, we want to find the proportions of the number of equal pairings by looking at the ratio of cell pairs of equal type and pair of any type. We denote the cardinality, i.e. the number of elements, of a set *S* by $\vert S \vert$. Thus, we calculate the proportions $r_{uu}(k)$ of $u^+v^-$ pairs at distance *k* as well as the proportions $r_{vv}(k)$ of $u^-v^+$ pairs at distance *k* via

64 $$\begin{aligned} r_{uu}(k) = \frac{\vert S^u_k\vert }{\vert S_k\vert } \quad \text{ and }\quad r_{vv}(k) = \frac{\vert S^v_k\vert }{\vert S_k\vert }. \end{aligned}$$

The goal is to relate these proportions with the probability of randomly picking two cells of equal type. For this, we need the total number of $u^+v^-$ cells $T^u$ and $u^-v^+$ cells $T^v$. The chance of picking one $u^+v^-$ cell is $\vert T^u\vert$. If one has already been picked, then the remaining chance of picking a second one becomes $(\vert T^u\vert -1)/(N-1)$. In total, we can write the probability of randomly selecting two $u^+v^-$ cells or two $u^-v^+$ cells as

65 $$\begin{aligned} p_{uu} = \frac{\vert T^u\vert (\vert T^u\vert -1)}{N(N-1)} \quad \text{ and }\quad p_{vv} = \frac{\vert T^v\vert (\vert T^v\vert -1)}{N(N-1)}. \end{aligned}$$

Combined, the PCFs measure the ratios of $u^+v^-$ or $u^-v^+$ cell pairs within every possible distance normalized by the probability of finding these cell pairs, i.e.

66 $$\begin{aligned} \rho _u(k)&= \frac{r_{uu}(k)}{p_{uu}} = \frac{\vert S^u_k\vert N(N-1)}{\vert S_k\vert \vert T^u\vert (\vert T^u\vert -1)}, \end{aligned}$$

67 $$\begin{aligned} \rho _v(k)&= \frac{r_{vv}(k)}{p_{vv}} = \frac{\vert S^v_k\vert N(N-1)}{\vert S_k\vert \vert T^v\vert (\vert T^v\vert -1)}. \end{aligned}$$

For a uniformly distributed amount of $u^+v^-$ or $u^-v^+$ cells, the correlation function returns a value close to 1 for every cell distance *k*. Consequently, deviations from 1 yield information about how much more or fewer equal cell pairs are found in certain ranges.

## Numerical results

In this section, we present the numerical solutions of (18). The explicit Euler method is used to solve the ODE until a steady state is reached. We consider two different types of signaling. Paracrine signals that exhibit low diffusivity can be described by a nearest neighbor signal. For larger diffusivities, the signal disperses throughout the tissue such that its intensity decreases with the distance traveled.

### Nearest neighbor signaling

#### Signal construction

A signal that is secreted by one cell and diffuses slowly throughout the tissue will likely end up only affecting neighboring cells. Similar to Bessonnard et al. (2014), Stanoev et al. (2021), we investigate a signal that gets activated by *u* and depends on the neighboring cells. However, rather than investigating a time resolved signal, we consider it only as an instantaneous response such that

68 $$\begin{aligned} s_i = \frac{1}{\vert N_G(i)\vert }\sum _{j \in N_G(i)} u_j. \end{aligned}$$

Here, we used the notation $N_G(i)$ from graph theory to denote the neighbors of vertex *i* in the graph *G*. We can also write the whole signal in terms of an adjacency matrix. For (68), this matrix is

69 $$\begin{aligned} A = (A_{i,j})_{i,j=1,...,M}, \text{ with } A_{i,j} = {\left\{ \begin{array}{ll} \frac{1}{\vert N_G(i)\vert } &{} \text {if } j \in N_G(i)\\ 0 &{} \text {if } j \notin N_G(i) \end{array}\right. }. \end{aligned}$$

The signal can ultimately be written as ${\varvec{s}} = A{\varvec{u}}$. From the steady state (24) we know $u_i = 0$ for some of the cells in a heterogeneous tissue. Therefore, the minimum of the signal will also be 0. The non-zero steady state has a rough upper bound, hence

70 $$\begin{aligned} u_i = \frac{r_u}{\gamma _u} - \frac{1+\eta _s s_i}{\eta _u} < \frac{r_u}{\gamma _u}. \end{aligned}$$

Therefore, the maximum signal also obeys

71 $$\begin{aligned} \max _i s_i = \max _i \left( \frac{1}{\vert N_G(i)\vert }\sum _{j \in N_G(i)} u_j \right) < \frac{1}{\vert N_G(i)\vert }\sum _{j \in N_G(i)} \frac{r_u}{\gamma _u} = \frac{r_u}{\gamma _u}. \end{aligned}$$

Using parameter combinations, such that

72 $$\begin{aligned} \frac{r_u}{\gamma _u} \gg \frac{1 + \eta _s \frac{r_u}{\gamma _u}}{\eta _u}, \end{aligned}$$

transforms the upper bound into a proper estimate of the signal values, such that we can conclude

73 $$\begin{aligned} \min _i s_i = 0 \quad \text{ and }\quad \max _i s_i \approx \frac{r_u}{\gamma _u}. \end{aligned}$$

Hence, the stability interval can be approximated by

74 $$\begin{aligned} -\Delta \varepsilon _v + \ln \left( \frac{r_v\gamma _u}{r_u\gamma _v}\right)< & {} -\Delta \varepsilon _u < -\Delta \varepsilon _v + \ln \left( 1 + e^{-\Delta \varepsilon _s-\Delta \varepsilon _{vs}} \frac{r_u}{\gamma _u}\right) + \ln \left( \frac{r_v\gamma _u}{r_u\gamma _v}\right) .\nonumber \\ \end{aligned}$$

#### Pattern formation

**Fig. 5:**
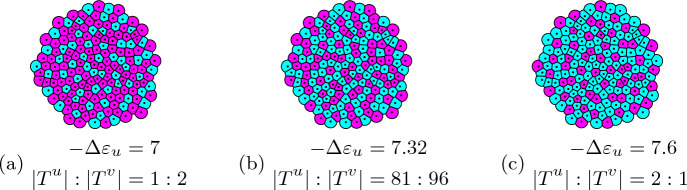
Checkerboard pattern for three different ratios of $u^+v^-$ and $u^-v^+$ cells. The coloring uses the cell’s expression levels for *v*. High *v* expressions are colored in magenta, low *v* expressions (high *u*) in cyan (color figure online)

Models of cell differentiation characterized by lateral inhibition tend to form an approximate checkerboard pattern of cells (Collier et al. 1996) with a trend towards alternating cell types wherever possible. In fact, the term “lateral inhibition” comes from the fact that cells of a primary cell fate prevent cells in the environment from adopting the same fate. Despite the name, the signal between neighboring cells in Collier et al. (1996) is activating rather than inhibiting. Our model differs by the inclusion of mutual inhibition and auto-activation. Thus, the goal in this section is to show that our model is still capable of forming checkerboard patterns. The parameter values and initial conditions used in any of the following simulations can be found in Table S2. The remaining energy difference $-\Delta \varepsilon _u$ is varied based on (56) to influence the cell type ratio. In the resulting cell fate pattern, $u^+v^-$ cells mostly avoid other $u^+v^-$ cells in their neighborhood as much as the given proportion enables them to do so (Fig. 5, see Fig. S1 for temporal evolution). Increasing $-\Delta \varepsilon _u$ increases the proportions of $u^+v^-$ cells throughout the tissue. This results in more and more $u^+v^-$ cells with neighbors of equal type. Increasing the proportions even further leads to the opposite effect of $u^-v^+$ cells trying to avoid being neighbored to cells of the same type. In total, both cell types try to avoid cells of equal type in their neighborhood as much as the proportions and the geometry of the tissue allows. In a regular $8\times 8$ grid with, this would resemble a checkerboard. Hence, the term checkerboard pattern.

**Fig. 6:**
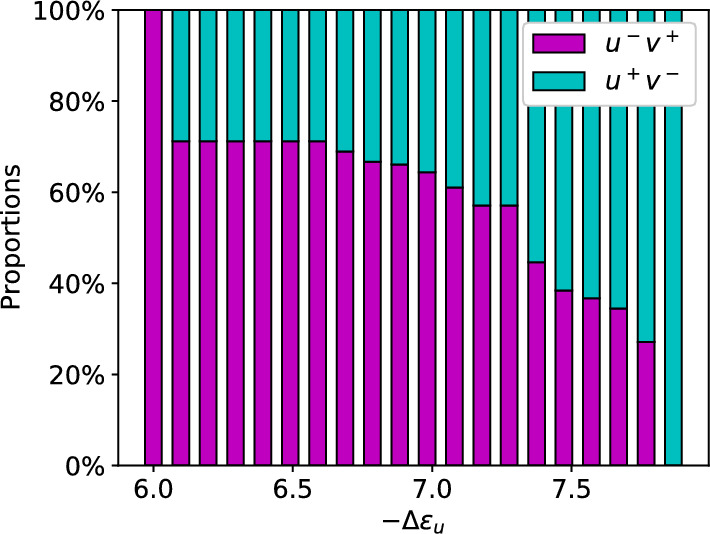
Simulated cell type proportions for 20 equidistant values of $-\Delta \varepsilon _u$ spanning over the stability interval (56). Cell proportions for $u^+v^-$ are colored in cyan, $u^-v^+$ in magenta

#### Cell type proportions

In some biological systems, it might be crucial to generate cell types in precise proportions like in the mouse embryo (Saiz et al. 2016, 2020). Hence, we are interested in exploring the capabilities of the model to create certain proportions. The range of possible cell type proportions can be found in the stability interval (56). For the parameter combinations chosen in this study, we get

75 $$\begin{aligned} \frac{1 + \eta _s \frac{r_u}{\gamma _u}}{\eta _u} \le 0.0043 \ll 0.1 = \frac{r_u}{\gamma _u}. \end{aligned}$$

Hence, our approximation for the stability interval (74) is valid and yields the following parameter restrictions for the heterogeneous steady states:

76 $$\begin{aligned} \eta _u \in (403.43, 2606.08) \qquad \Longleftrightarrow \qquad -\Delta \varepsilon _u \in (6,7.87). \end{aligned}$$

The various cell type proportions (Fig. 6) were simulated by dividing the bounding interval (76) into 20 equidistant values for $-\Delta \varepsilon _u$. The simulation results underline the result of the stability analysis. At the left and right boundaries, we achieve homogeneity. In between, increasing $-\Delta \varepsilon _u$ yields a monotonous transition from only $u^-v^+$ to only $u^+v^-$ cells. The boundary regions suggest that proportions with about $73\%$ of one cell type and $27\%$ of the other are the maximum and minimum cell proportions achievable before reaching homogeneity. An analytical analysis of the relation of the cell type proportions and the parameter $-\Delta \varepsilon _u$ reveals why these jumps occur. Focusing again on a single cell in the tissue, we already identified the tipping point of the cell’s fate via Eq. (29). Deviating from $s=s^*$ to $s>s^*$ will increase the binding probability for *v*, tipping its fate towards $u^-v^+$. Analogously, $s<s*$ will lead to $u^+v^-$. By definition, $s_i$ is the mean of a cells neighboring $u_j$ values. Assuming the neighbors to be in steady state and using the steady state approximation $u_i \approx r_u/\gamma _u$, the signal can be written as a fraction

77 $$\begin{aligned} s_i = \frac{l_i}{\vert N_G(i)\vert } \frac{r_u}{\gamma _u}, \qquad l_i \in \{0,..., \vert N_G(i)\vert \}, \end{aligned}$$

where $l_i$ denotes the number of $u^+v^-$ cells adjacent to cell *i*. From this, we can determine the maximum number of $u^+v^-$ cells in a neighborhood for the cell to still adopt the fate $u^+v^-$. Therefore, we replace $s_i$ with $s^*$ and solve the equation for $l_i$ to find

78 $$\begin{aligned} l_i = \vert N_G(i)\vert \frac{\gamma _v}{r_v} \frac{\eta _u - \eta _v}{\eta _v\eta _s \eta _{vs}}. \end{aligned}$$

Since we are looking for a natural number, the final result is

79 $$\begin{aligned} l^{\max }:= \left\lfloor \vert N_G(i)\vert \frac{\gamma _v}{r_v} \frac{\eta _u - \eta _v}{\eta _v\eta _s \eta _{vs}}\right\rfloor . \end{aligned}$$

Here, $\lfloor x \rfloor$ describes the floor function, i.e. the nearest lower integer of a number *x*. Small differences between energy coefficients $\eta _u = \eta _v + \delta$ with $\delta > 0$ being small, will lead to $l^{\max } = 0$. Therefore, a single $u^+v^-$ cell will have no neighbor of equal type. At the same time, cells without any received signal, i.e. $s_i=0$ will adopt $u^+v^-$ fate (Fig. 2). In conclusion, a cell surrounded only by $u^-v^+$ cells will adopt $u^+v^-$ fate, whereas a cell with a single $u^+v^-$ in its neighborhood has to adopt $u^-v^+$ fate. On an ideal hexagonal grid, i.e. each cell has exactly six neighbors, an ideal arrangement would amount to 1/3 of the cells being $u^+v^-$. This estimate nearly fits the simulated proportion jumps of $27\%$ at both ends. An exact number cannot be determined, as the number of neighbors varies from cell to cell with an average of $5.5 \pm 1000000$ neighbors. Further increases of $\eta _u$ only lead to discrete increases of $l^{\max }$, explaining the different jumps in cell type proportions.

### Distance-based signaling

#### Signal construction

Motivated by recent studies of long ranging paracrine signaling (Fiorentino and Scialdone 2022; Stanoev et al. 2021) we investigate the effect of a distance-based signaling on the pattern formation and cell type proportion. Depending on how a signal disperses in space, not only directly neighboring cells can have an impact on a cell’s fate. It is possible, that the collective effect of cells that are further away might also influence its fate decision. Again, the secreted signal of a cell is activated by $u_i$. We define the received signal $s_i$ as the weighted sum of secreted signals over all other cells and obtain

80 $$\begin{aligned} s_i = \left( \sum _{j\ne i}s_j q^{d_{ij}-1}\right) \bigg /\left( \max _k\sum _{j\ne k} q^{d_{kj}-1}\right) , \qquad q \in [0,1]. \end{aligned}$$

**Fig. 7:**
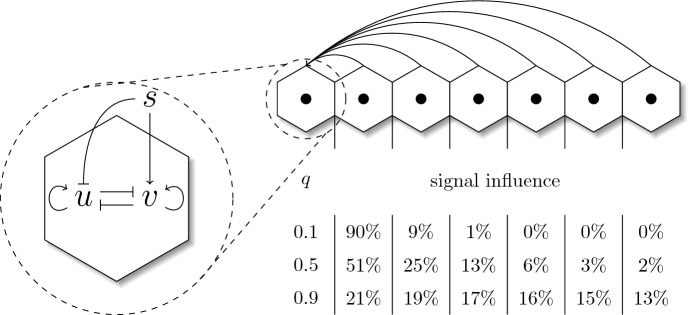
Illustration of the GRN represented by our model as well as a representative example of the signaling in a one-dimensional cell line. Inside the cell, *u* and *v* mutually inhibit each other. Additionally, *v* gets activated by an extracellular signal, whereas *u* is inhibited by the same. The signal received by the first cell on the left of the line is the sum of all cell–cell communication between one cell and any other cell in the system. The table highlights how much each cell contributes to the received signal for different dispersions $q \in \{0.1,0.5,0.9\}$. Percentages are rounded to the nearest integer

Here, we use the distances $d_{ij}$ from our cell graph. The weights $q^{d_{ij}-1}$ define the fraction of the signal that gets transported from cell to cell. Let e.g. $q=0.1$, then second nearest neighbors of a cell receive only $10\%$ of the signal of the direct neighbors (Fig. 7). The denominator in (80) is used for normalization. It describes the weights of the cell that gets the highest possible signaling weights. In a perfectly arranged circular tissue, this would be the cell right in its center due to the mean of cell distances $d_{ij}$ being lower. The dispersion parameter *q* enables us to describe the transition from a direct neighbor signal to an equally dispersed signal. For $q=0$, the weights become

81 $$\begin{aligned} q^{d_{ij}-1} = 0^{d_{ij}-1} = {\left\{ \begin{array}{ll}1, \quad \text {for } d_{ij}=1\\ 0, \quad \text {for } d_{ij}>1 \end{array}\right. }. \end{aligned}$$

Hence, the weights for all cells that are not directly in contact with the respective cell are 0 and we obtain a mechanism similar to the local signal (68). Alternatively, $q=1$ yields

82 $$\begin{aligned} q^{d_{ij}-1} = 1^{d_{ij}-1} = 1. \end{aligned}$$

This describes the case of every cell having the same impact on other cells independent of the distance between them. In summary, there is a continuous transition from a next neighbor signal at $q=0$, through a distance-based global signal for $q \in [0,1]$, to an evenly distributed signal at $q=1$. In matrix representation we get

83 $$\begin{aligned} A = (A_{i,j})_{i,j=1,...,M} \text{ with } A_{i,j} = {\left\{ \begin{array}{ll} a q^{d_{ij}-1} &{} \text {if } i \ne j\\ 0 &{} \text {if } i = j \end{array}\right. } \end{aligned}$$

and the normalization factor

84 $$\begin{aligned} a = \left( \max _k\sum _{l\ne k} q^{d_{kl}-1}\right) ^{-1}. \end{aligned}$$

For the estimation of the stability interval, we again use the upper bound $u_i < r_u/\gamma _u$, such that

85 $$\begin{aligned} s_i&< \frac{r_u}{\gamma _u}\left( \sum _{j\ne i} q^{d_{ij}-1}\right) \bigg /\left( \max _k\sum _{j\ne k} q^{d_{kj}-1}\right) \end{aligned}$$

86 $$\begin{aligned}&\le \frac{r_u}{\gamma _u}\left( \max _k \sum _{j\ne k} q^{d_{kj}-1}\right) \bigg /\left( \max _k\sum _{j\ne k} q^{d_{kj}-1}\right) = \frac{r_u}{\gamma _u}. \end{aligned}$$

At this point, we realize that the estimation follows the exact same procedure as before, leading to (74). It should be mentioned, that the choice of (80) is not based on a physical foundation, but is simply a mathematical construct that could be easily unified with the result of the stability analysis but still allows to investigate the concept of different signaling ranges.

#### Pattern formation

**Fig. 8:**
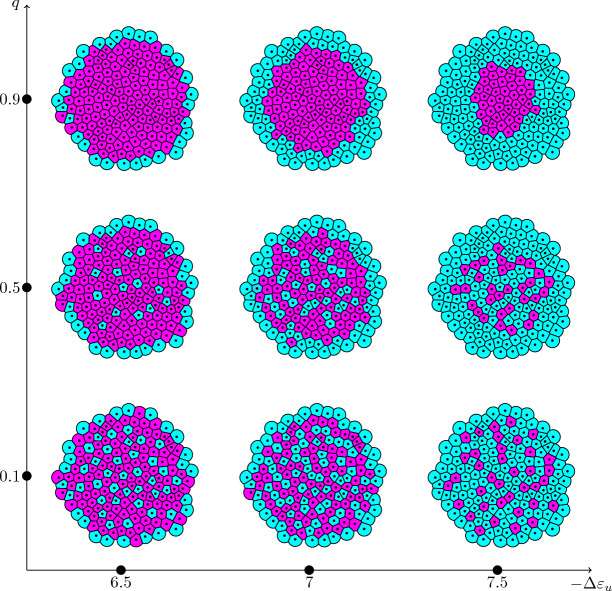
Different patterns generated by the model on a tissue geometry with 177 cells. Colors depict the values of $v_i$ in steady state. High values of $v_i$ correspond to low values in $u_i$ and vice-versa, i.e. cyan and magenta represent $u^+v^-$ and $u^-v^+$ cells, respectively. From left to right, $-\Delta \varepsilon _u$ increases. From bottom to top, the dispersion *q* increases (color figure online)

We want to investigate the effect of the distance-based signal on the formation of the patterns. Therefore, we showcase nine simulation results of organoids with different cell type proportions and different signal dispersions (Fig. 8 and Fig. S2-S4). The patterns generated for $q=0.1$ can mostly be considered of the checkerboard type. In contrast to the averaged nearest neighbor signal, the signal in this case is not averaged over the number of neighbors. Cells at the boundary typically have three to four neighboring cells, whereas cells in the bulk area have a mean of six neighbors. Therefore, cells at the boundary will potentially not be able to get the same amount of signal as cells in the bulk area. Such boundary effects are representative of fixed size systems such as embryoid bodies or organoids. Furthermore, they are likely to occur at tissue-tissue or tissue-cavity interfaces within developing embryos.

The received signal however, is the deciding factor with regard to the cell fate decision in our model. The low amounts of signal received at the boundary make them more likely to adopt the $u^+v^-$ fate. As *q* increases, we see a higher accumulation of $u^+v^-$ cells near the boundary with a slight clustering behavior in the bulk. For $q=0.9$, the signal disperses strongly enough to generate an engulfing pattern, where $u^-v^+$ cells are completely surrounded by $u^+v^-$ cells. Varying the proportions of $u^+v^-$ cells via $-\Delta \varepsilon _u$ has a different effect based on the signal dispersion *q*. For low *q* and increasing $u^+v^-$ proportions, we find a ring of $u^+v^-$ cells engulfing the remaining checkerboard pattern. For medium to large *q* this effect is more strongly pronounce with the formation of several layers of $u^+v^-$ cells engulfing the tissue.

**Fig. 9:**
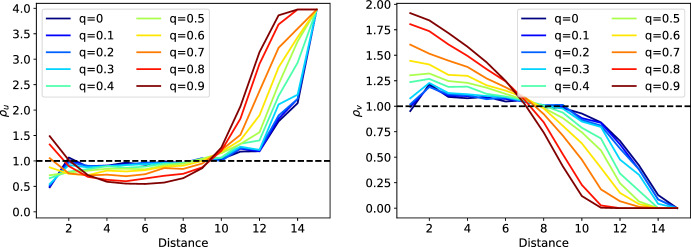
PCFs for $u^+v^-$ cells (left) and $u^-v^+$ cells (right) for different dispersion parameters *q*. Any PCF represents a tissue with a ratio of $u^+v^-$:$u^-v^+= 88:89$. The dashed black line at 1 resembles the PCF values of an ideal uniform distribution of two different cell types. If values lie above 1, this means there are more pairs found at that distance. Consequently, values below 1 resemble fewer pairs

The pattern formation with respect to *q* can be quantified using the PCFs for both $u^+v^-$ and $u^-v^+$ cells (Fig. 9). For comparison, we used a bisection on the stability interval to find values for $-\Delta \varepsilon _u$ that lead to a ratio of 89 : 88 $u^+v^-$ and $u^-v^+$ cells for every single *q*. We discover that an increase in *q* leads to a decrease of $\rho _v$ for large distances, i.e. less and less pairs of $u^-v^+$ cells pairing in the boundary regions. Simultaneously, it increases for small distances due to the cells accumulating in the center. For $\rho _u$, we see a slight increase for large *q* for small distances and a tremendous one for large distances for all *q*. The slight increase at small distances comes from the fact that the $u^+v^-$ cells arrange in layers at the boundary. The values for intermediate distances slightly decrease as the corresponding regions become more and more devoid of $u^-v^+$ pairs. In conclusion, a distance-based signal according to (80) generates patterns ranging from checkerboard to engulfing by increasing the dispersion parameter *q*. Additionally, the PCFs capture the characteristics of these patterns, making it a powerful tool for pattern identification and comparison.

#### Cell type proportion

**Fig. 10:**
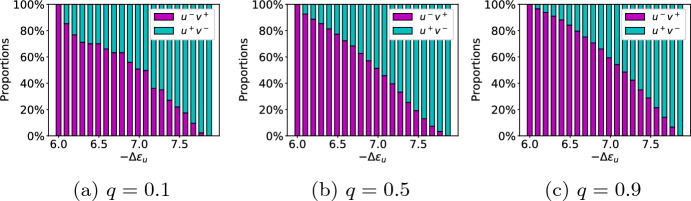
Simulated cell type proportions with respect to $-\Delta \varepsilon _u$. Simulations were performed by dividing the stability interval for $-\Delta \varepsilon _u$ into 20 equidistant values. Dispersion parameter *q* increases from **(a)** to **(c)** resulting in different scenarios

For different dispersion parameter values *q*, the proportions of $u^-v^+$ show a monotonous decrease with increasing energy difference $-\Delta \varepsilon _u$ (Fig. 10). For low values of *q*, the proportions show some similarities to the local model due to individual larger jumps (Fig. 10a). These jumps become less pronounced for medium (Fig. 10b) and high dispersions (Fig. 10c). Altogether, we have established full control over the cell type proportions.

## Discussion

In this study, we have derived and analyzed a model that allows us to generate cell differentiation patterns based on a system of mutual inhibition of two TFs, auto-activation and cell–cell communication. The model was thoroughly analyzed and simulated patterns were characterized.

### Derivation of the model from statistical mechanics

Statistical mechanics has already proven its usefulness in biological model systems like ion channel opening and closing as well as oxygen hemoglobin binding (Garcia et al. 2011). These ideas have further been investigated for transcriptional regulation and were successfully applied for a wide variety of examples (Bintu et al. 2005a, b). To our knowledge, cell fate decision models have not been combined with statistical mechanics to date. We derived a specific model based on two mutually inhibiting TFs *u* and *v* with auto-activation and an external signal inhibiting *u* and activating *v*. Assuming that auto-activation is the dominant factor in transcriptional regulation, we assume that RNA polymerase binding corresponds to the binding of *u* and *v*, respectively. Based on this, we were able to derive binding probabilities of RNA polymerase to the respective promoter. A system of ordinary differential equations was generated by combining these probabilities with constant production rates and exponential decay. As long as the auto-activation remains unchanged, minor changes in the GRN such as the removal of either the signal activation or the signal inhibition can still be managed by adjusting the equations accordingly.

### Analysis of the model allows accurate determination of the stability of heterogeneous steady states

On the single cell level, we identified that the received signal determines the fate of a cell. There is a critical value of this signal, such that the cell will adopt $u^-v^+$ fate if this value is undercut, or $u^+v^-$ fate if the value is exceeded. This leads to the signal being the relevant factor of the switching behavior in this system. This describes a different point of view compared to systems that utilize differences in initial conditions to generate a cell fate switch (Cherry and Adler 2000; Huang et al. 2007). At the same time, models that incorporate a signal dependency, have not yet been analyzed in such great detail (Bessonnard et al. 2014; Stanoev et al. 2021; Tosenberger et al. 2017). Exact expressions for all possible steady states were derived. A stability analysis enabled us to identify parameter values, such that only the states corresponding to $u^+v^-$ and $u^-v^+$ fates are stable. Thus, we were able to limit the system to these two cell fates. On the level of multiple cells, we found analytical expressions of parameter bounds guaranteeing heterogeneous steady states. This means that within these bounds the pattern created by the system will always be a mixture of two different cell types. In conclusion, we have provided the necessary analytical tools to guarantee the generation of heterogeneous patterns of two different cell types.

### Averaged nearest neighbor signaling leads to checkerboard patterns

In some biological systems, cell communication is hypothesized to be limited to direct neighbors. An example for this is the lateral inhibition of Delta and Notch in epithelial tissue of *Drosophila*, which has been studied in great detail (Collier et al. 1996). A different example is found in the preimplantation development of the mouse embryo, where TFs NANOG and GATA6 decide the fate of cells in the inner cell mass. Different computational studies have investigated the effects of activation by an external signal in this biological system (Bessonnard et al. 2014; Mot et al. 2016; Tosenberger et al. 2017). A great common feature in all of these systems is the formation of checkerboard patterns, i.e. patterns in which cells of one type minimize the number of equal neighbors. Fittingly, we also found this type of pattern in our simulations using an averaged nearest neighbor signaling. We took this one step further and analyzed the possible cell type proportions one can create using this model. An analytical expression for the maximum number of equal cell types in a cell’s neighborhood tells us that the cell type proportions are highly linked to the average number of neighboring cells in the system. In our 2D simulations cell type proportions below $30\%$ and above $70\%$ are not possible.

### Distance-based signaling enables a range of patterns from checkerboard to engulfing

In addition to the nearest neighbor signal, we investigated the effects of a signal that is capable of being dispersed throughout the tissue. This global cell–cell communication enables a range of patterns. From two cell types in a checkerboard like arrangement to one cell type engulfing the other depending on the signal dispersion. The introduced dispersion parameter *q* allows us to artificially vary between a signal that only reaches the neighboring cells and a signal that spreads evenly in the tissue. Simulations have shown that for low signal dispersion $u^+v^-$ and $u^-v^+$ cells tend to avoid being adjacent to the same cell type, hence we again recovered the checkerboard pattern. Furthermore, when increasing the signal dispersion, $u^+v^-$ cells accumulate more at the boundary such that overall larger clusters of equal cell types are formed. High signal dispersion leads to an ideal segregation of cells with $u^+v^-$ engulfing $u^-v^+$ cells. Engulfing patterns are often believed to be the result of differential adhesion of two cell types. Indeed, it has already been demonstrated that the minimization of the energy as a function of differential adhesion leads to this type of engulfing (Emily and François 2007). Not only have we found an alternative way to generate these patterns, but at the same time we were able to unify the formation of both checkerboard and engulfing patterns under the notion of differently dispersing signals.

### Biological examples for the different patterns

The lateral inhibition principle exemplified by the Notch-Delta signalling pathway leads to alternating fates between neighboring cells. At the multicellular level, this results in a checkerboard pattern. Different systems show patterns of longer spatial scales of local clusters such as the developing mouse neural tissue (Hawley et al. 2022) or in vitro stem cell differentiation in embryoid bodies (White et al. 2013) and ICM organoids. Completely engulfing patterns have been observed as transient patterns in differentiating embryoid bodies (White et al. 2013) and ICM organoids that have been cultured for a sufficiently long time (Mathew et al. 2019). Partly engulfing patterns arise in different embryos including mammalian, e.g. mouse or human (Płusa and Piliszek 2020), and fish, e.g. zebrafish (Gilbert 2014). The relevance of our model results to these different systems has to be evaluated based on careful comparison of the arising patterns and underlying GRNs.

### Conclusion

We have provided a new model to describe transcriptional regulation for a system of mutually exclusive TFs. Furthermore, the model was analyzed in great detail with respect to parameters and stability. The model was extended by signaling mechanisms describing the cell–cell communication. The local and global signaling obey a simple mathematical rule depending on the number of cells it has to travel across in order to reach its destination. A detailed description of the signaling transport mechanism, possibly including diffusion and advection mechanisms, provides room for further research. Additionally, signal production and uptake of cells play a crucial role in how effective different means of signal transport might be. Another perspective can be achieved by incorporating cell growth and cell division into the model and analyzing their effect on the resulting patterns. With this in mind, our study paves the way for numerous subsequent studies regarding signal-based pattern formation in developmental systems.

### Supplementary Information

Below is the link to the electronic supplementary material.

Supplementary file 1 (pdf 860 KB)

## Data Availability

Not applicable.
