# Supplementary material for: Adjusting the range of cell–cell communication enables fine-tuning of cell fate patterns from checkerboard to engulfing
Source: J Math Biol. 2023 Sep 7;87(4):54. doi: 10.1007/s00285-023-01959-9 (PMC10485129; doi:10.1007/s00285-023-01959-9)
Supplement: Supplementary file 1 — (pdf 860 KB) [file 285_2023_1959_MOESM1_ESM.pdf]

Supplementary Materials

Model variable	Description
u	Dimensionless concentration of TF U
v	Dimensionless concentration of TF V
s	Dimensionless concentration of signal S

Table S1: List of model variables and their descriptions.

Model parameter	Fixed value	Description
$-\Delta\varepsilon_u$	$\in (6, 7.87)$	Energy difference w.r.t. binding of u
$-\Delta\varepsilon_v$	6	Energy difference w.r.t. binding of v
$-\Delta\varepsilon_s$	2	Energy difference w.r.t. binding of s
$-\Delta\varepsilon_{vs}$	2	Energy difference w.r.t. binding of v and s
r_u	1	Transcription rate of u
r_v	1	Transcription rate of v
γ_u	10	Decay rate of u
γ_v	10	Decay rate of v
q	$\in (0, 1)$	Dispersion parameter for distance-based signal
u_0	$\frac{3}{4}\frac{r_u}{\gamma_u}\left(1+\frac{\xi}{10}\right)$	Initial condition for u and $t = 0$
v_0	$\frac{3}{4}\frac{r_v}{\gamma_v}\left(1+\frac{\xi}{10}\right)$	Initial condition for v and $t = 0$
ξ	$\sim N(0, 1)$	Standard normal distribution noise
T	1000	Simulation time
Δt	0.2	Time steps

Table S2: List of model parameters and initial conditions together with their values and descriptions. Parameters were chosen arbitrarily with the aim to generate visually distinguishable cell fates.

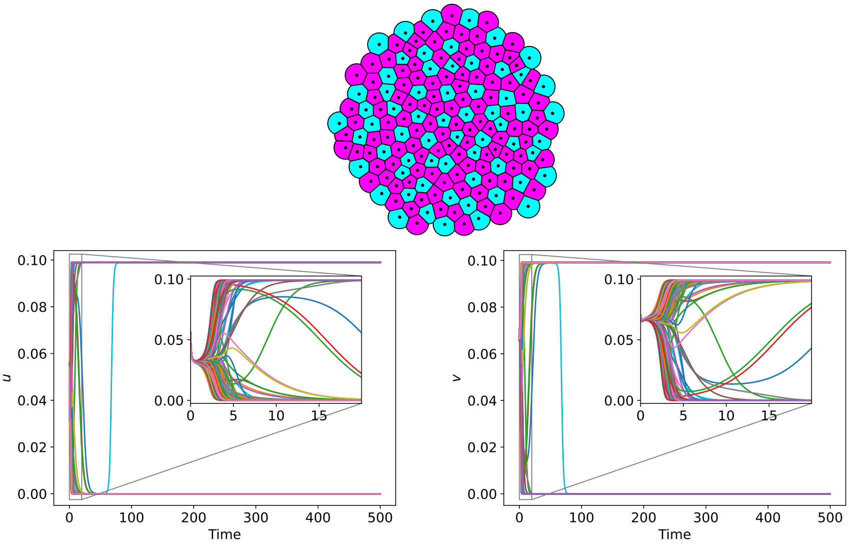

(a) Time evolution of u

(b) Time evolution of v

Figure S1: Examplary time evolution of u (left) and v (right) for the organoid above using nearest neighbor signaling. Each of the 177 lines represent a single cell. Zoomed in portion highlights the earlier stage up to $T = 20$. The energy difference was chosen as $-\Delta\varepsilon_u = 7$.

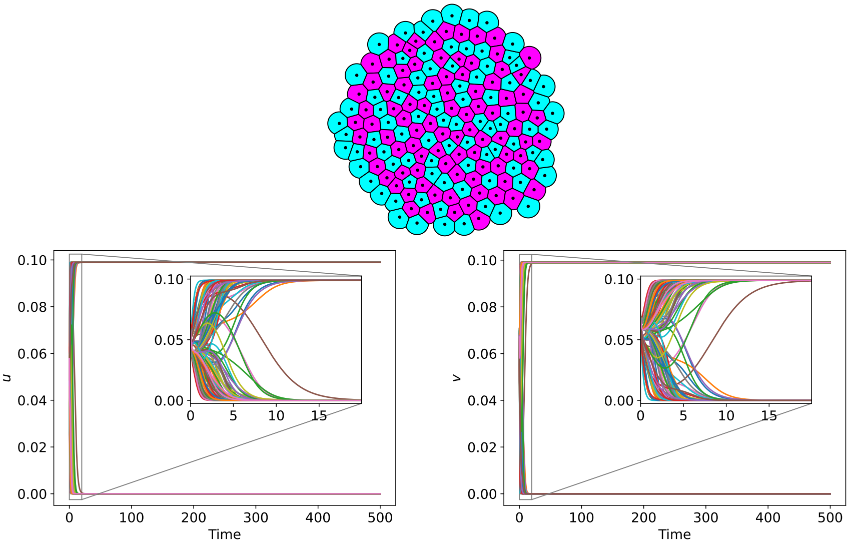

(a) Time evolution of u (b) Time evolution of v

Figure S2: Examplary time evolution of u (left) and v (right) for the organoid above using distance-based signaling with dispersion parameter $q = 0.1$. Each of the 177 lines represent a single cell. Zoomed in portion highlights the earlier stage up to $T = 20$. The energy difference was chosen as $-\Delta\varepsilon_u = 7$.

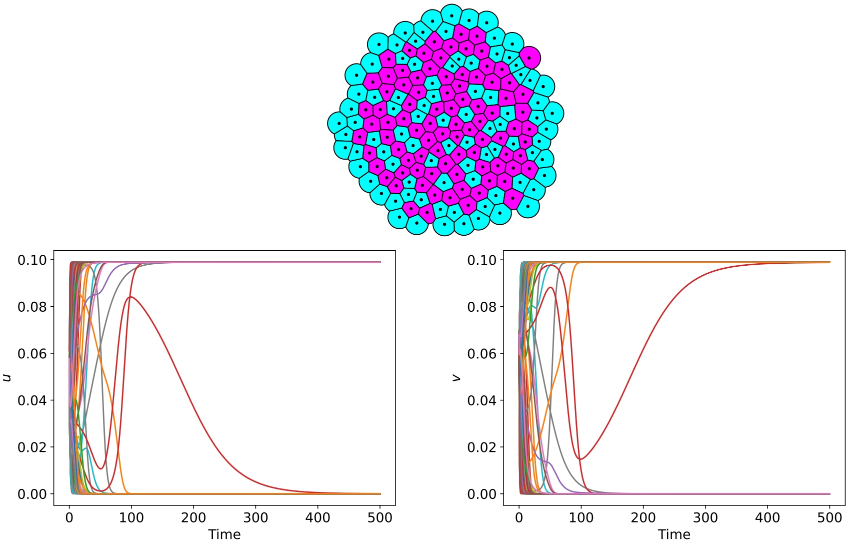

(a) Time evolution of u (b) Time evolution of v

Figure S3: Examplary time evolution of u (left) and v (right) for the organoid above using distance-based signaling with dispersion parameter $q = 0.5$. Each of the 177 lines represent a single cell. The energy difference was chosen as $-\Delta\varepsilon_u = 7$.

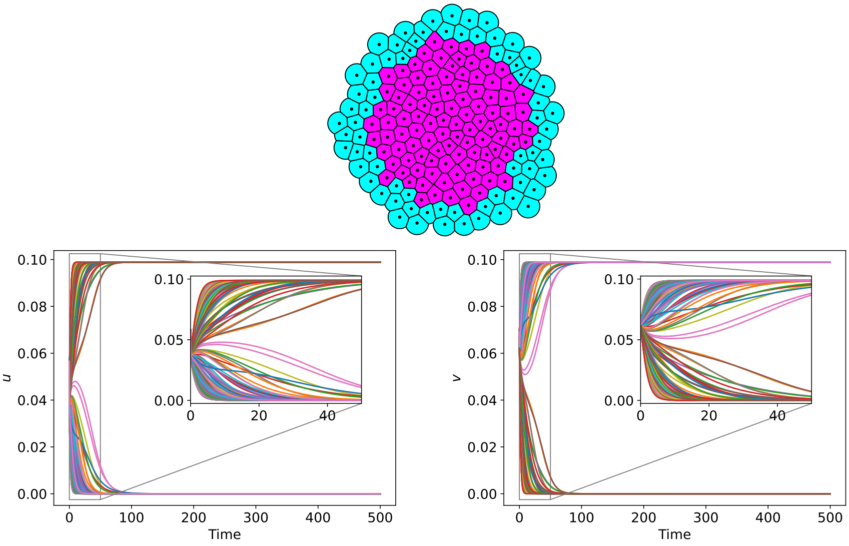

(a) Time evolution of u (b) Time evolution of v

Figure S4: Examplary time evolution of u (left) and v (right) for the organoid above using distance-based signaling with dispersion parameter $q = 0.9$. Each of the 177 lines represent a single cell. Zoomed in portion highlights the earlier stage up to $T = 20$. The energy difference was chosen as $-\Delta\varepsilon_u = 7$.
